# Tuberculosis in an Allogeneic Transplant Kidney: A Rare Case Report and Review of Literature

**DOI:** 10.7759/cureus.11661

**Published:** 2020-11-23

**Authors:** Sreethish Sasi, Manoj K Varghese, Arun P Nair, Samar Hashim, Muna Al Maslamani

**Affiliations:** 1 Internal Medicine, Hamad Medical Corporation, Doha, QAT; 2 Infectious Diseases, Hamad Medical Corporation, Doha, QAT

**Keywords:** renal transplant, tuberculosis, immunosuppression

## Abstract

Tuberculosis (TB) is a common post-transplant infection with high prevalence in developing countries due to reactivation. Post-transplant TB involves the respiratory system in 50% of patients, followed by disseminated involvement in 30%. The risk of tuberculosis of renal allograft post-transplantation is determined by disease endemicity in the donor population and the immunosuppressant regimen. TB can cause allograft rejection and graft loss due to delayed diagnosis or reduced immunosuppressant drug efficacy. A 23-year-old lady was seen 40 days after cadaveric unrelated renal transplantation from China. She was on immunosuppression with tacrolimus, mycophenolate, and prednisolone. Examination showed low-grade fever and infected surgical site in the right iliac fossa draining pus. Imaging showed fluid pockets, parenchymal micro-abscesses, and perinephric collections in the right iliac fossa communicating with skin. A diagnosis of renal allograft TB without dissemination was made after TB polymerase chain reaction (PCR) from early morning urine was positive. She was started on anti-TB therapy. The sinus tract healed, and renal parameters improved after six months of therapy. Follow-up magnetic resonance imaging (MRI) showed resolution of the micro-abscesses as well as the surrounding fluid collection. Renal angiogram demonstrated well-perfused, normally functioning, non-obstructed renal transplant. Tuberculosis of renal allograft should be considered in a transplant recipient with pyrexia of unknown origin and persistent discharge from the surgical site, not responding to antimicrobials. Tuberculosis of transplant kidney can cause graft loss due to allograft rejection when there is a delayed diagnosis, or as anti-TB drugs reduce the efficacy of immunosuppressant medications. The index of suspicion should be high when donor status is unknown or if the donor is from an endemic tuberculosis area. Timely diagnosis and treatment helped to save the transplanted kidney of our patient without rejection.

## Introduction

Tuberculosis (TB) is a common post-transplant infection with high prevalence in developing countries due to reactivation. The prevalence of post-transplant TB is 3.1% to 15% in Asia, 1.5% to 3.5% in the Middle East, 1.7% to 5% in Europe, and 1.5% in the United States [1]. TB in transplant recipients is either due to reactivation of latent infection in the recipient, unrecognized infection in the allograft, or acquisition of new infection post-transplantation [1]. Post-transplant tuberculosis involves the respiratory system in 50% of the cases followed by disseminated involvement in 30%, lymph nodes in 5%, skin and soft tissue in 4%, the genitourinary system in 4%, intestine in 3%, the central nervous system in 2%, and bones in 1%. In 16% of the cases, it presents as pyrexia of unknown origin (PUO) [2]. The prevalence of post-transplant renal TB is quite low, and only a few cases have been reported. In a study by Torre-Cisneros et al., the median onset of TB was 183 days after transplantation (range 28-499 days). Donor-derived TB occurs earlier than reactivation of latent TB in the recipient [3]. Symptoms of post-transplant TB depend on the type of solid organ transplanted. Fever, night sweats, and weight loss occur in the majority of the patients [1]. TB should be considered in all transplant recipients with unexplained fevers, night sweats, and weight loss. Diagnosis may require invasive procedures such as bronchoscopy or biopsy.

Renal TB occurs by mycobacterial seeding of the urogenital tract via hematogenous spread. Renal parenchymal lesions, including interstitial nephritis and glomerulonephritis, rarely occur, resulting in granulomas that heal by fibrosis or rupture into the tubule years later excreted in the urinary tract resulting in the spread of infection [4]. Common symptoms are persistent pyuria, microscopic hematuria, low back pain, increased urinary frequency, and urgency. Systemic symptoms, such as fever and weight loss, occur less frequently [5]. The diagnosis is established by demonstrating tubercle bacilli in the urine by culture or urine polymerase chain reaction (PCR). Positive urine acid-fast stain is not diagnostic for TB since nontuberculous mycobacteria may be present [6]. Radiographic imaging, in the form of computed tomography (CT), high-resolution ultrasound, or magnetic resonance imaging (MRI), is indicated for patients with suspected renal TB [4]. Radiographic evidences strongly suggestive of TB are strictures in the collecting system, asymmetric caliectasis, calcification, and hydronephrosis [4].

## Case presentation

A 23-year-old lady was seen in the infectious diseases’ clinic, 40 days after a renal transplant for routine evaluation. She had end-stage renal disease (ESRD) because of unknown cause and was on continuous ambulatory peritoneal dialysis (CAPD) for two years. The donor's kidney was from an unrelated cadaveric source in China, whose personal information and medical history were unknown. The patient was human leukocyte antigen (HLA)-antibody negative, and her immunological risk status was determined to be low/moderate risk. She was not given any initial induction therapy and was started on triple immunosuppressant maintenance therapy with tacrolimus, mycophenolate mofetil (MMF), and prednisolone. She was on antimicrobial prophylaxis with valganciclovir and trimethoprim-sulfamethoxazole. The immediate post-operative period was complicated with urinary tract and wound infections, treated with a short course of intravenous ampicillin-sulbactam, showing a good response. There were no sick contacts or household exposure to pets. The only recent travel was to China for transplantation. Physical examination showed that she was febrile, but other vital signs were stable. Local inspection of the surgical site showed an infected wound in the right iliac fossa draining pus. There was tenderness on palpation over the surgical site. Systemic examination was otherwise unremarkable. There was no organomegaly or free fluids in the abdomen. The hernial orifices were intact.

Her initial laboratory evaluation results were unremarkable except for elevated urea, creatinine, and C-reactive protein (Table 1). Pus culture from surgical wound culture grew candida species, while urine showed *Citrobacter braakii* and extended-spectrum beta-lactamase (ESBL) resistant *Escherichia coli*. Serology for cytomegalovirus (CMV) showed a PCR titer of 194 IU/ml. Quantiferon test for tuberculosis was negative twice, before and after transplant. She was treated with a course of ertapenem 1 gram daily for seven days and anidulafungin 100 milligrams daily for two weeks. However, she continued to have a low-grade fever and persistent pus discharge from the wound, even though the repeated urine and wound cultures were negative.

**Table 1:** Table showing results of relevant laboratory tests on initial presentation µL, microliters; gm/dL, grams/deciliter; µmol/L, micromoles per liter; U/L, International units per liter; mg/dl, milligrams per deciliter; mg/L, milligrams per liter; ng/ml, nanograms per milliliter.

Detail	Results	Normal Range
White Blood Cells (x10^3^/µL)	6.2	4-10
Absolute Neutrophil Count (ANC)%	86	55-70
Lymphocytes%	6.9	20-40
Monocytes%	5.1	2-8
Eosinophils%	0.4	1-4
Basophils%	0.6	0.5-1
Platelets (x10^3^/µL)	220	150-400
Hemoglobin (gm/dL)	10	12.0-15.0
Urea (mmol/L)	9.96	2.5-6.7
Creatinine (µmol/L)	107	50-98
Total Bilirubin (µmol/L)	13.7	3.4-20.5
Alkaline Phosphatase (U/L)	69.4	40-150
Alanine Aminotransferase (U/L)	23	0-55
Aspartate Aminotransferase (U/L)	23	5-34
FK-506 Level (ng/mL)	13.2	7-21
C-Reactive Protein, CRP (mg/L)	80	0-5
Procalcitonin (ng/ml)	0.14	<0.5

**Figure 1:**
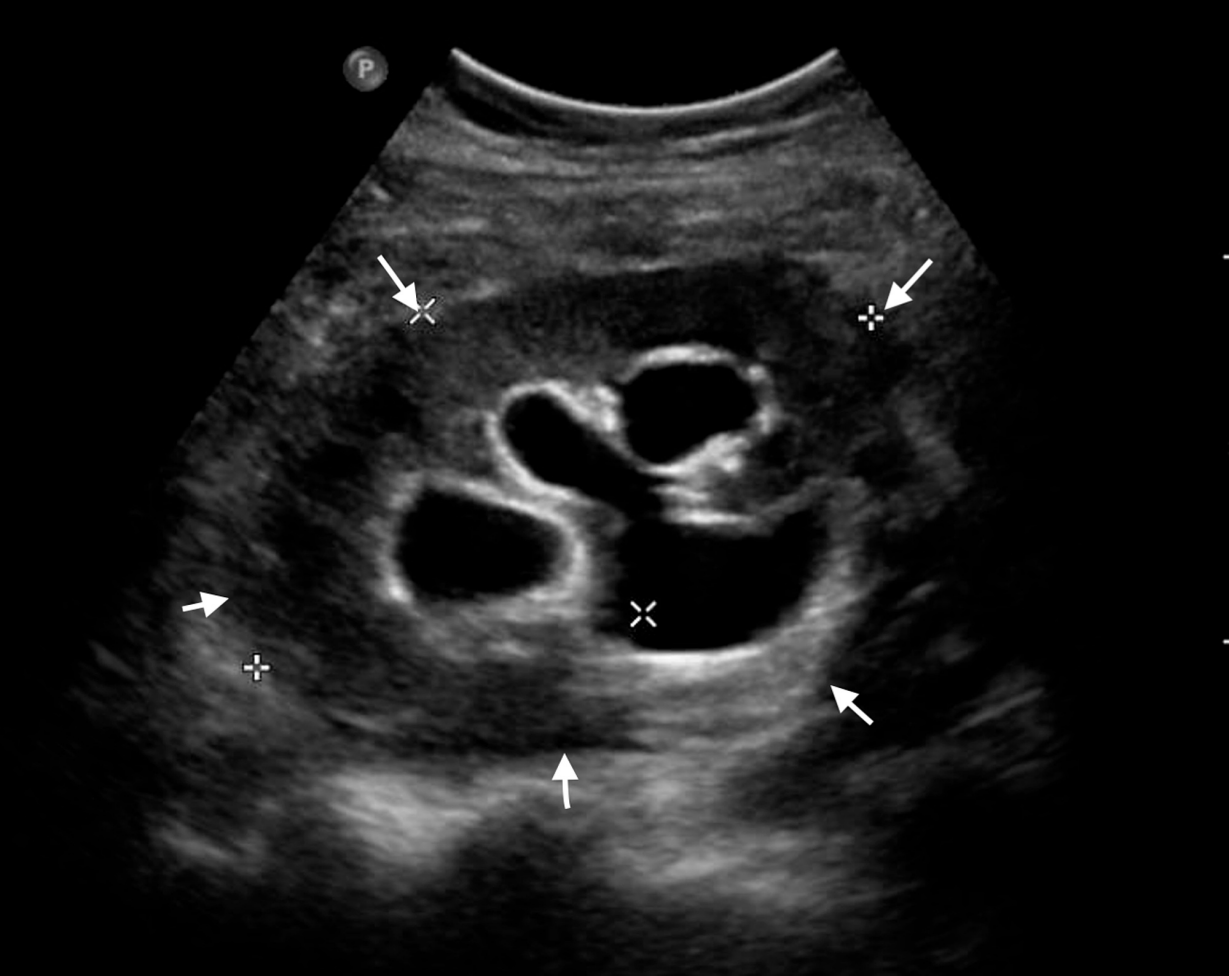
Ultrasound of transplanted kidney Transplanted kidney is seen in the right iliac fossa measuring 9.4 cm x 5.9 cm x 5.4 cm with volume of 160 ml (white arrows). It shows normal echogenicity and cortical thickness with mild hydronephrosis and trace of perinephric fluid. No evidence of anastomotic stenosis. Duplex parameters are all within normal limits.

The initial ultrasound of the transplanted kidney showed normal echogenicity and cortical thickness without any peri-graft collections. There was no evidence of any anastomotic stenosis, and all duplex parameters were normal. Mild hydroureteronephrosis of the transplanted kidney with suboptimal distention of urinary bladder was seen. No definite calculus was identified. The same imaging was repeated after two weeks as there was persistent pus discharge from the surgical site. It showed two echogenic foci of size 4 mm in the lower pole of the transplanted kidneys, representing parenchymal calcifications or stones. Multiple, small, poorly localized fluid pockets were noted in the right iliac fossa along the subcutaneous plane of the surgical site, largest measuring 1.5 cm x 0.7 cm. In conclusion, there were new-onset parenchymal calcifications associated with peri-graft collection communicating with the outside as sinus (Figure 1).

**Figure 2:**
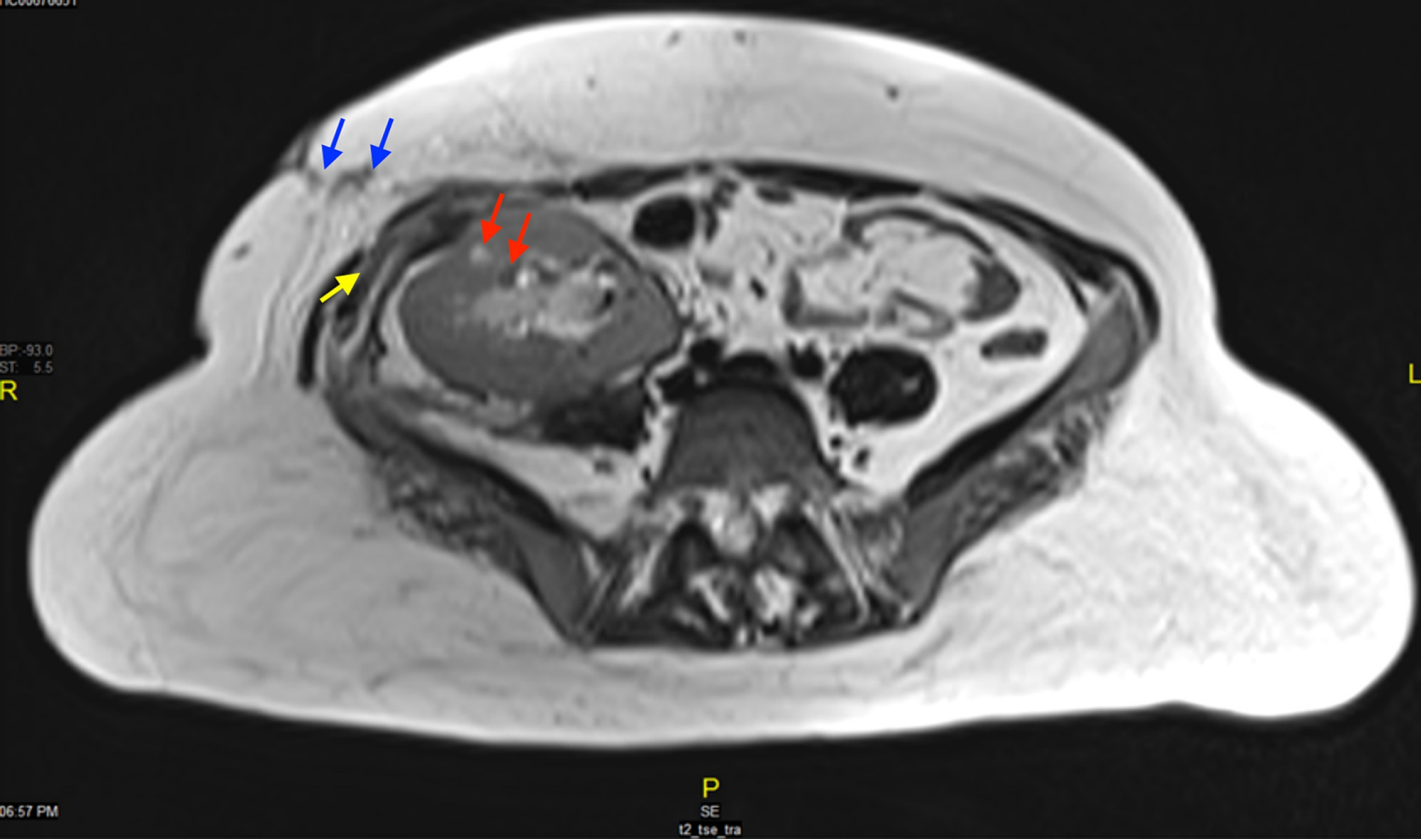
Magnetic resonance imaging (MRI) of the abdomen and pelvis The figure shows heterogeneous pockets of fluid collections along the posterior aspect of the transplanted kidney (red arrows), tracking along the lateral aspect into the right anterior pelvic wall (blue arrows), which contains another collection measuring approximately 4 cm x 1.5 cm (yellow arrow), with surrounding extensive inflammatory changes and possible external opening. The non-localized collection posterior to the transplanted kidney measures approximately 4.5 cm x 1.2 cm and demonstrates restricted diffusion suggesting abscess.

**Figure 3:**
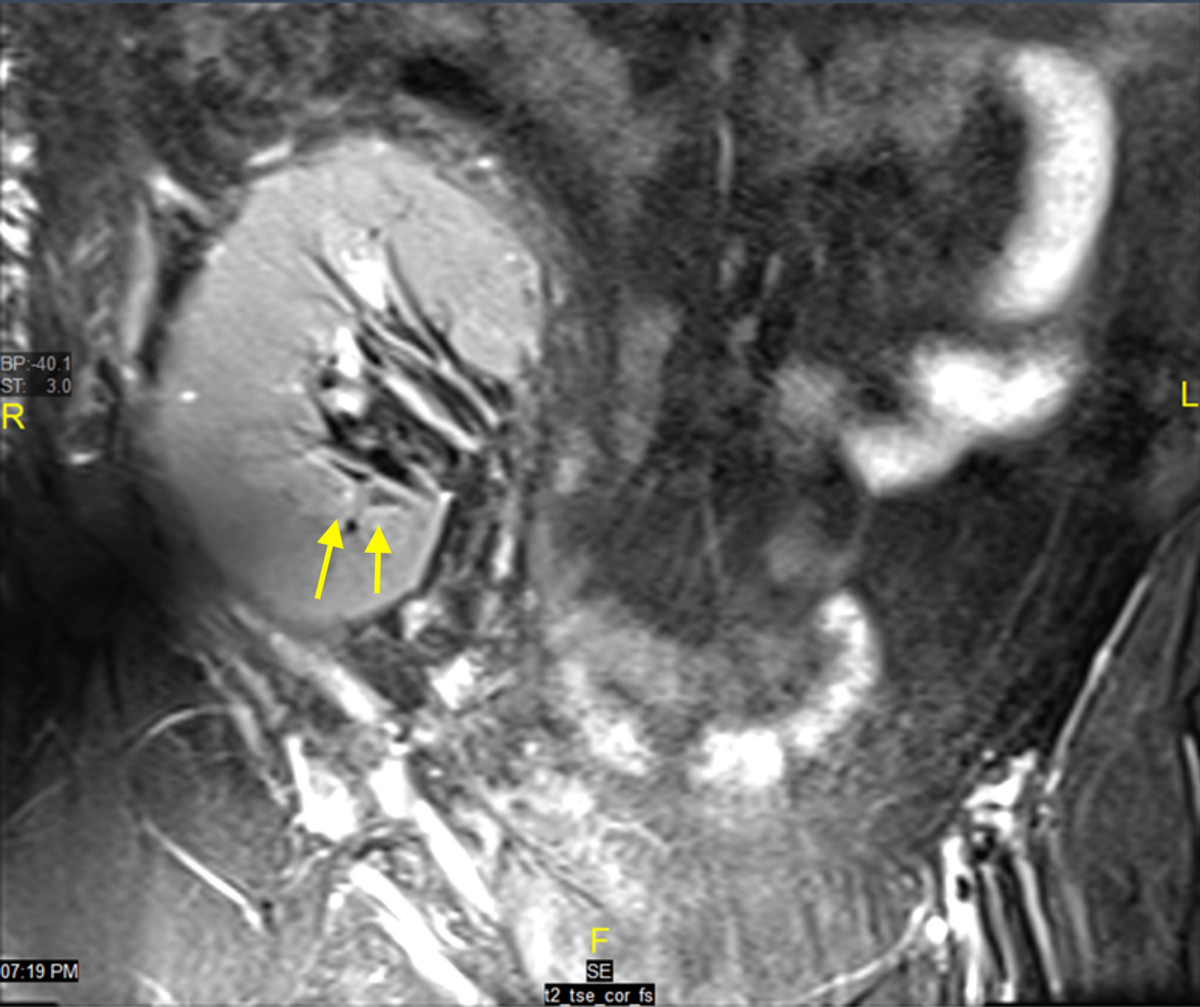
Magnetic resonance imaging (MRI) of the abdomen and pelvis The figure shows mild to moderate hydroureteronephrosis with the presence of calyceal clubbing and tiny cystic areas around the calyces, especially in the lower pole (yellow arrows). Multiple small parenchymal T2 bright cystic regions were also identified with few such areas demonstrating restricted diffusion, suggesting parenchymal micro-abscesses. Differential diagnosis of the possible etiology is graft site candidiasis, actinomycosis, mycobacterium tuberculosis, *Citrobacter braakii*, and cytomegalovirus (CMV).

CT of the abdomen and pelvis showed the transplanted kidney with multiple stones and surrounding perinephric fat stranding. MRI of the abdomen showed mild to moderate hydronephrosis of the transplanted kidney with parenchymal micro-abscesses. There was a poorly localized perinephric collection with pockets along the posterior aspect of the transplanted kidney extending into the subcutaneous fat with surrounding edema and a cutaneous opening (Figures 2, 3). 

Microscopy of the smear from the pus revealed a Ziehl-Neelsen stain-positive organism, with 2500 acid-fast bacilli (AFB) per 100 fields. TB PCR was also positive from the discharge, and the mycobacterium was rifampicin sensitive. Two sets of early morning urine samples were positive for TB PCR. Chest x-ray was normal, and TB workup from sputum was negative. A diagnosis of renal tuberculosis in allogeneic renal transplant kidney without dissemination was made. Anti-TB therapy was immediately started with rifampicin 150 mg, isoniazid 75 mg, pyrazinamide 400 mg, ethambutol 250 mg, and pyrazinamide 40 mg daily. The therapeutic levels of tacrolimus were regularly monitoring, targeting 3-7 ng/mL, and its dose was gradually increased from 6 to 10 mg (in two divided daily doses), considering interaction with rifampicin. There was a regular follow-up, and significant clinical improvement was seen after six months of therapy. The sinus tract healed, and renal parameters normalized. Repeated MRI abdomen revealed resolution of the micro-abscesses and surrounding fluid collection. A renal angiogram showed a well-perfused, normally functioning, non-obstructed transplant kidneys.

## Discussion

Genitourinary tuberculosis is the second most common form (20% to 40%) of extra pulmonary tuberculosis in developing countries and third most common in developed countries. The prevalence of TB of the transplanted kidney is quite low (<4%) [1]. Genitourinary TB in kidney transplant patients commonly occurs secondary to reactivation in the setting of immunosuppression. In such cases, kidneys get involved as part of disseminated TB. In rare cases where TB involves renal allograft alone without dissemination, it is donor-derived and related to the transplanted organ [7]. A systematic search of PubMed, Scopus, and Google Scholar for case reports of isolated tuberculosis of renal allografts without disseminated involvement published in the last 20 years revealed that renal allograft TB without disseminated involvement was reported in six cases, summarized in Table 2 [8-11]. Our case had the shortest time frame from transplant to the diagnosis of allograft TB. Two of the cases were diagnosed by urine TB PCR, whereas four others were diagnosed by biopsy of transplant kidney.

**Table 2:** Summary of case reports of isolated tuberculosis of renal allografts without disseminated involvement published in the last 20 years compared to our case Tx, transplantation; AFB, acid-fast bacilli; TB, tuberculosis; PCR, polymerase chain reaction; CsA, cyclosporin; AZA, azathioprine; MMF, mycopheolate mofetil; Tac, tacrolimus; US, ultrasound; CT, computed tomography; MRI, magnetic resonance imaging; PUO, pyrexia of unknown origin; M, male; F, female; CGN, chronic glomerulonephritis; DN, diabetic nephropathy; PKD, polycystic kidney disease; HRZE, isoniazid (H), rifampicin (R), pyrazinamide (Z), and ethambutol (E).

S. No.	Author	Year	Sex/Age	Donor Type	Country of Tx	Indication for Tx	Immunosuppressant Used		Time From Tx to Diagnosis of Allograft TB	Clinical Presentation	Diagnosis					Treatment	Outcome
							Induction	Maintenance			Urine			Imaging	Biopsy		
											AFB Smear	TB PCR	TB Culture				
1	Khaira et al. [8]	2004	F/30	Live unrelated (Husband)	India	Unknown	No	CsA, AZA steroids	40 months	PUO	-ve	-ve	-ve	US of transplant kidney showed necrotizing granulomas	Positive AFB smear from granulomas	HZE Ofloxacin	Cured
2	Khaira et al. [8]	1997	M/55	Live unrelated (Wife)	India	Unknown	No	CsA, AZA steroids	120 months	PUO	+ve	+ve	+ve	None	None	HZE Ofloxacin	Cured
3	Khaira et al. [8]	1995	M/36	Live related (Father)	India	CGN	No	CsA, AZA steroids	84 months	PUO	-ve	-ve	-ve	None	Granulomatous interstitial nephritis	HRZE	Improved
4	Al-Nesf et al. [9]	2005	F/53	Live unrelated	Philippines	DN	No	CsA, MMF steroids	2 months	PUO	-ve	-ve	-ve	US, CT, and MRI abdomen showing large lymphocele surrounding transplant kidney	Graft biopsy showing caseating granuloma, with AFB seen on Z-N staining. lymphocele	HRZE	Failure with allograft nephrectomy
5	Malone et al. [10]	2003	M/53	Cadaveric unrelated	Ireland	Adult PKD	No	Tac, MMF steroids	29 months	Nausea, worsening of GFR	+ve	+ve	+ve	None	None	HRZE	Cured
6	Siu et al. [11]	2000	M/39	Cadaveric unrelated	China	CGN	No	CsA, MMF steroids	3 months	PUO	-ve	-ve	-ve	US of graft kidney was grossly normal, and CT of the abdomen showed mild ascites only	Granulomatous inflammation of the interstitium with epithelioid cells and lymphocytes. Ziehl–Nielsen stain was positive for AFB.	HRZE	Failure with allograft nephrectomy
7	Our Case	2016	F/23	Cadaveric unrelated	China	Unknown	No	Tac, MMF steroids	1.5 months	PUO, Non-healing surgical wound	+ve	+ve	+ve	Hydronephrosis and echogenic collections with cutaneous opening	Not done	HRZE	Cured

Immunosuppression following solid organ transplants increases the chance of contracting TB. This complicates the post-transplant period as the antibiotics used to treat TB interact with immunosuppressants and cause toxicities. TB can cause transplant rejection and increase overall morbidity. The disease endemicity in the population and the type of immunosuppressant regimen determine tuberculosis incidence after renal transplantation [12]. Tuberculosis of transplant kidney can cause graft loss due to allograft rejection when there is a delayed diagnosis, or as anti-TB drugs reduce the efficacy of immunosuppressant medications. Agarwal et al. showed that the use of tacrolimus compared to cyclosporine significantly decreases post-transplant TB [13]. Apart from immunosuppressants, other risk factors include diabetes mellitus, chronic liver disease, use of corticosteroids more than 10 milligrams/day, or high-dose pulse therapy. The number of rejection episodes and the duration of hemodialysis are also associated with an increased incidence of tuberculosis [12]. Rifampicin increases the catabolism of glucocorticoids and tacrolimus as it is a potent CYP3A4 isoenzyme inducer, thus decreasing their serum levels. The dose requirement of tacrolimus may be increased up to 10 times when used with rifampicin [14]. It reduces mycophenolate mofetil action by induction of mycophenolic acid (MPA) glucuronidation and possibly rifampin-associated alterations in multidrug resistance-associated protein 2 (MRP2)-mediated transport of mycophenolic acid glucuronide (MPAG) and acyl-MPAG [11]. Quantiferon and other interferon gamma release assays (IGRAs) cannot be used to exclude latent tuberculosis infections (LTBI) in chronic immunosuppressive therapy [9]. A meta-analysis of 709 patients from four randomized controlled trials showed that isoniazid prophylaxis reduces the risk of TB in post-transplant patients, without an increase in hepatitis. However, there is a marked discrepancy among national renal transplant units within the United Kingdom (UK) in pharmacologic prophylaxis for TB and the selection of individuals for this treatment [15]. MRI is a sensitive modality for demonstrating features of renal TB, including tissue edema, asymmetric perinephric fat stranding, and thickening of Gerota's fascia, all of which may be clues to focal pyelonephritis of tuberculous origin [4]. In our case, the transmission of TB from the donor is suggested by the presence of micro-abscesses in the transplanted kidney, which ruptured involving the perinephric tissue, followed by communication with the exterior in the form of a sinus draining pus. At the same time, our patient did not have any recognized risk factors for TB, including previous exposure, malnutrition, or living in an endemic area. TB transmission from the donor should be suspected when there is no evidence of inactive tuberculosis in the pre-transplant workup, and risk factors for getting tuberculosis post-transplant are eliminated. Both of these risk factors were not present in our patient. The diagnosis of TB was made in the first 40 days post-transplantation suggests that it may be donor origin.

## Conclusions

Many cases of PUO post-renal transplant are due to TB of the engrafted kidney. However, such cases are underdiagnosed and seldom reported. TB of the renal allograft should be considered in any post-transplant patient with PUO and persistent discharge from the surgical site not responding to conventional antimicrobials. A high index of suspicion should be entertained if the donor TB status is unknown or if the donor is from an endemic area of tuberculosis. Microcalcifications in the MRI of the transplanted kidney indicate healed TB granulomas, which may be re-activated in the setting of immunosuppression. A timely intervention in the form of diagnosis and treatment can help prevent graft loss and further morbidity.
